# Plasmonic Nano-bolas Hunt DNA Targets

**DOI:** 10.1021/acsnanoscienceau.5c00131

**Published:** 2025-11-14

**Authors:** Aura Cencini, Graziano Rilievo, Mirco Zerbetto, Mary Bortoluzzi, Federica Tonolo, Fabio Vianello, Alessandro Cecconello, Massimiliano Magro

**Affiliations:** † Department of Comparative Biomedicine and Food Science, 9308 University of Padova, Viale dell’Università 16, 35020, Legnaro (PD), Italy; ‡ Department of Chemical Science, 9308 University of Padova, via Marzolo 1, 35131, Padova (PD), Italy; § Department of Molecular and Translational Medicine, 9297 University of Brescia, Viale Europa 11, 25123, Brescia (BS), Italy

**Keywords:** DNA nanotechnology, self-assembly, gold nanoparticle, fluorescence, quenching-enhancing

## Abstract

We present the design principles and assembly route for a reconfigurable DNA-scaffolded nanomachine comprising a fluorophore and two gold nanoparticles (AuNPs) operated by DNA strand displacement. The mechanism confines the fluorophore in the proximity of one or simultaneously two DNA-tethered 15 nm AuNPs, resulting in discrete emission levels associated with the system state. Bi- and single-molecule DNA scaffolds were compared as alternative building blocks, aiming at the optimal structure in terms of reversibility, response to molecular triggers, and signal-to-noise ratio. Upon comparison, single-molecule DNA scaffold (i.e., nano-bolas), devoid of intrastructural equilibria, was only minimally affected by cross-talk interferences and stood out for its highly reversible transitions, lower noise, and better kinetics. Distance-dependent responses and kinetics were fully in harmony with theoretical modeling, well illustrating the nano-bolas interconversion between a linear and a quasi-ring geometry. The nano-bolas actuator could find application as an ultrasensitive, reversible, and small-volume plasmonic reporter for single-strand nucleic acid analytes.

## Graphical abstract

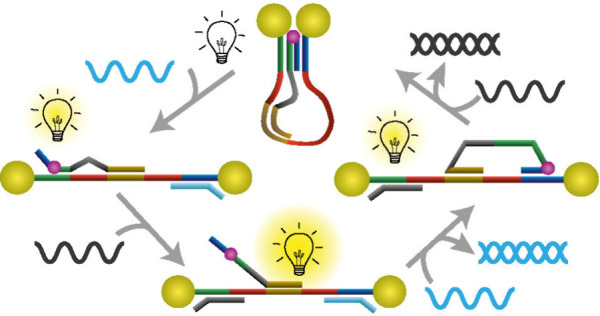

In the context of DNA nanotechnology, novel creative solutions are constantly being reported, demonstrating nucleic acids exceptional versatility as molecular building blocks. Thanks to their ability to store structural information in the base sequence, nucleic acids were used to fabricate a kaleidoscope of highly complex and functional supramolecular assemblies.^1–3^ In addition, when used as a static or dynamic scaffold, DNA allows the possibility of programming ligand binding,^4^ the positioning of nanoparticles in space,^5,6^ and, in general, the construction of self-assembled structures^7–9^ incorporating inorganic active species or displaying programmable mechanical responsiveness.^10,11^ Such programmable dynamic structures opened an avenue for DNA computing^12^ and storage systems.^13–15^

Although DNA itself does not possess intrinsic optical properties, its unique programmability had a substantial impact in the field of photonic nanomaterial design, guiding plasmonic nanoparticle organization into well-defined 2D and 3D assemblies^16–18^ with an unprecedented accuracy in light manipulation.^19^ In the construction of precisely regulated and coordinated dynamic photonic hybrid nanostructures, capable of maneuvering their individual components in space, AuNPs are the common choice^20^ as optical units since they can be combined with DNA through simple thiol-gold chemistry.^21–23^ In recent years, these hybrid nano-bioconjugate nanostructures represented the standard for fine-tunable molecular machines, allowing the comprehension and control of soft-matter interplays in nanomechanical materials,^24^ to fabricate chiral plasmonic nano-objects,^25^ as well as to produce biomimicking cell-like objects.^26^ For instance, dynamic gold nanostructures were presented as emulators of motorized machines at the nanoscale.^27,28^ The mechanical forces of DNA-gold nanomachines can be chemically triggered by light, pH, ions, temperature or strand-displacement reactions,^29^ using DNA scaffolds ranging from individual duplexes to origami or tiles.^7^

The possibilities offered by DNA functional scaffolds extended their range of applications from imaging label and targeted therapeutic tools in drug delivery, to photodynamic therapy.^7,30–36^ As an example, DNA origami nanorulers found application in super-resolution fluorescence microscopy as reliable distance measurement standards.^37^ Moreover, by the integration of AuNPs, DNA dynamic scaffolds can be designed to provide an optical signal output that responds to the presence of biomarkers through molecular assembly or disassembly, due to plasmonic coupling at close interparticle distances.^38,39^

Over a broad spectrum of pathologies^32,40^ DNA and RNA serve as informative diagnostic biomarkers helpful to provide customized treatment plans and monitor the progression of medical conditions.^33,34^ It is worth mentioning that traditional detection approaches such as Northern blotting, microarray hybridization, real-time quantitative PCR, and RNA sequencing still represent benchmark methods but can be complex, time-consuming, and expensive.^35,36,41–44^ Here, to conceptualize the design principle for the assembly of a feasible DNA-scaffolded fluorescent reporter, two alternative approaches were compared. Both consist of self-assembled, dynamic plasmonic-fluorescent nanoarchitectures controlled by strand-displacement reactions, expected to generate four conformations and associated levels of quenching effects. The modulation of molecular fluorescence is obtained via finely tuned quenching phenomena, dependent on the distance between a AuNP surface and a fluorophore.^45^

In the first case, a “nanofork” dynamic structure was developed with sulfo-cyanine 3 fluorophore (Cy3) in the middle of the two fork prongs, namely, two DNA single strands presenting two AuNPs at their ends. The chemically triggered bending of the prongs generated four discrete states (i.e. open, two symmetric half-closed states, or closed), resembling pliers motion. Each configuration was characterized by a distinct fluorescence emission intensity via plasmonic quenching effects. In the second nanomachine, a single-stranded DNA (ssDNA) molecular cursor was precisely shifted over a linear ssDNA rail, while the motion was induced through the binding of competitive DNA single strands added to the solution sequentially. The cursor moved to specific domains of the rail, generating four discrete configurations, and set specific distances between Cy3 and two AuNPs, tethered at both ends of the DNA rail.

To obtain precise nanomachine movements and, therefore, to precisely maneuver the AuNPs and Cy3 in space, toehold-assisted strand-displacement reactions were finely programmed based on sequence-specific duplex formation. The two nanoactuators were self-assembled through simple wet reactions, using DNA complementary sequences. It is worth mentioning that, thanks to their optical properties (ε_520_ = 3.6 × 10^8^ M^–1^ cm^–1^), AuNPs offer the advantage of facilitating the recognition and purification of the hybrid architecture via gel electrophoresis (*vide infra*).

A detailed description of the steps required to assemble the two nanosystems is presented in the Supporting Information (SI). Briefly, the conceptual difference between the two systems is that one scaffold is held together by a partial DNA duplex, hence subjected to an equilibrium with its dissociated form, while the other scaffold presents covalent bonds only. For the latter, its structural robustness is thought to prevent cross-talk interferences caused by intrastructural equilibria. Once the nanomachines were fabricated, the reconfiguration transitions were optically characterized recording fluorescence emission changes (λ_em_ = 565 nm) upon addition of a small excess of the triggering strand (for additional details on the reconfiguration operations, including volumes and concentrations of all mixtures, see the SI). Fluorescence intensity was used as an indirect measurement of the reaction evolution (i.e. monitoring of the reaction kinetics), being the result of the contributions from the progressively disappearing initial state (the reagent) and the developing final state (the product).

The system performances were studied at a nanoreporter concentration of 0.5 nM. The first self-assembled structure comprises two single-DNA-functionalized 15 nm AuNPs, i.e. monovalent particles. This was obtained through thiol-gold chemistry using Cy3-modified oligonucleotide (1) or oligonucleotide (2) that were −SH modified at their 3′- or 5′-end, respectively. Electrophoretic separation and purification were carried out to isolate the nano-bioconjugate carrying one oligonucleotide. Figure 1a shows a scheme of the HS-DNA functionalization process along with an exemplary gel electrophoretic separation of the DNA-functionalized 15 nm AuNPs. Bands corresponding to bare AuNP reference and (1)- or (2)-functionalized AuNPs are indicated by arrows. From the band densities and the disappearance of the reference AuNP band, a ca. 100% yield of AuNP functionalization was estimated. The hybrids were then collected in a dialysis tube, upon band excision from the agarose gel, and cleaned in TBE buffer (additional details of the AuNP modification, separation, and purification are reported in the SI). Figure 1b shows a scheme of the dynamic DNA structure self-assembly. The two AuNP-DNA hybrids self-assembled in solution through a sequence-specific interaction, resulting in the (1)/(2) “fork” structure, in which a partial DNA duplex is the handle and the remaining ssDNA portions are rigidified by assisting strands (3) and (4) that release the corresponding hairpin and are similar to fork prongs, presenting the AuNPs at their ends. All DNA sequences are reported in Table S1.

**Figure 1:**
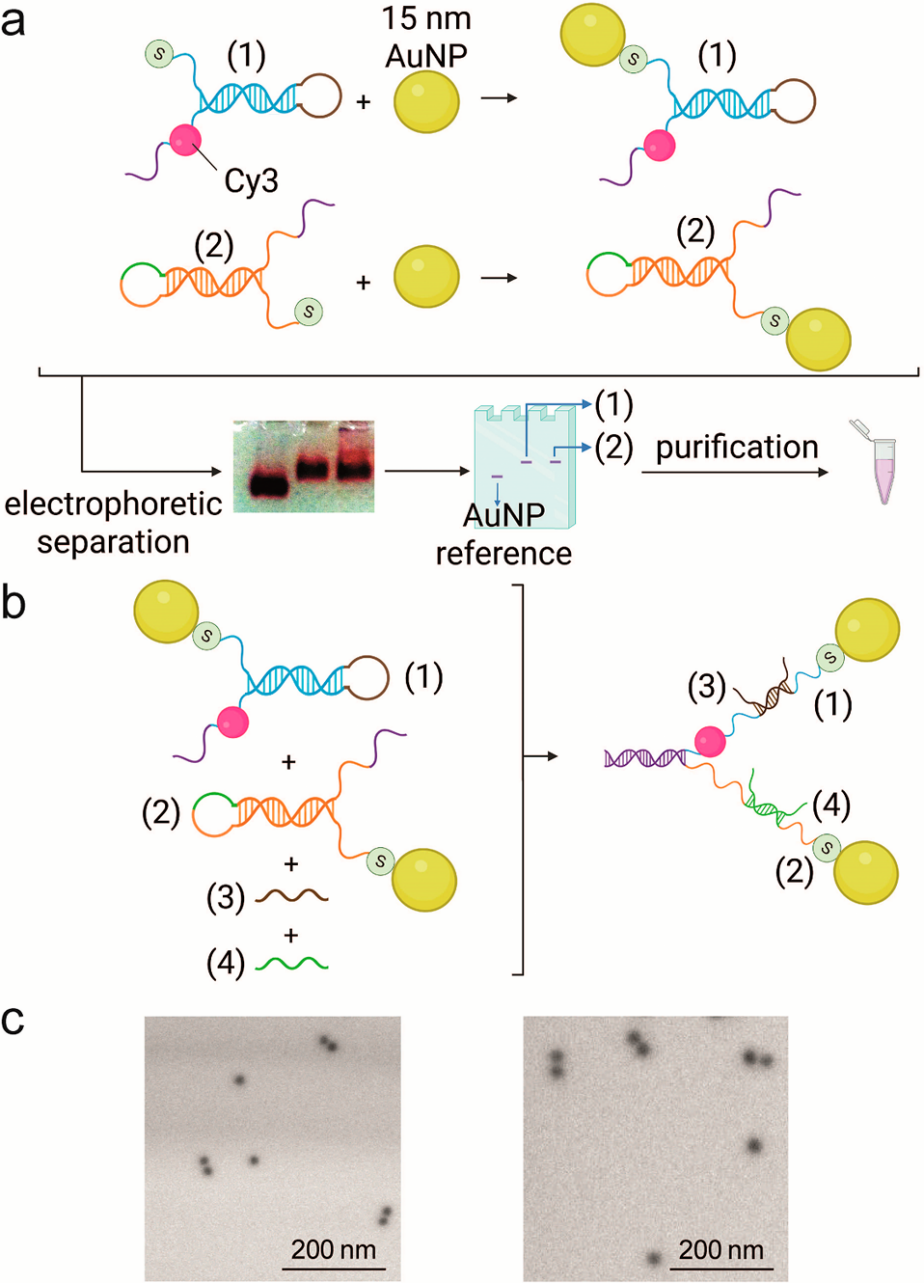
DNA fork fabrication scheme. (a) Functionalization of 15 nm AuNPs with single thiolated ssDNA and representative agarose gel electrophoretic separation showing bands corresponding to (1)- or (2)-functionalized AuNPs and bare AuNPs. (b) Sequence-specific self-assembly scheme of the fork DNA scaffold; strands (1) and (2) partially interact through their ends to form a duplex structure (the handle), whereas the remaining ssDNA portions (the prongs) are stabilized by rigidifying strands (3) and (4). (c) Representative STEM micrographs of the synthesized nanoassemblies in state F1.

In Figure 1(c), the synthesis products were morphologically characterized via scanning-transmission electron microscopy (STEM) where pairs of electrondense AuNPs were kept together by the DNA scaffold.

Cy3 fluorophore is set in a stationary position at the end of the duplex handle, whereas tethered AuNPs are maneuvered by a bending motion of the prongs, strands (1) and (2). AuNPs movement was triggered by the stepwise introduction in solution of strands (3′) and (4′) that form duplexes (3)/(3′) and (4)/(4′), upon removal of rigidifying strands (3) and (4), respectively. At this point, ssDNA portions of (1) and (2) assume thermodynamically more stable hairpin secondary structures that reduce the distance between the plasmonic surfaces and Cy3 and, therefore, modulate the quenching effect on the signal output. The structure can shift between four configurations, Figure 2(a): state F1 carrying blocking ssDNA (3) and (4) within partial duplexes (1)/(3) and (2)/(4), respectively. In this state, the quenching effect is at its minimum due to the large distance between Cy3 and each gold nanoparticle surface (ca. 11 nm). State F2 is realized by the displacement of strand (3) with strand (3′), resulting in (3)/(3′) duplex as byproduct, and positioning one AuNP close to Cy3 with an estimated distance equal to ca. 2 nm. Similarly, state F3 is realized by removal of the second blocking strand (4) using its respective complementary sequence (4′), determining the second AuNP approach to Cy3, at an estimated distance of ca. 2 nm. In this state, both AuNPs are confined in the proximity of Cy3, exerting their maximum quenching effect. Finally, state F4 is realized by addition of strand (3), which displaces the portion of strand (1) closer to the AuNP, opening the intramolecular duplex and regenerating duplex (1)/(3). Figure 2(b) shows a continuous fluorescence monitoring of the fork transitions across all states, while Figure 2(c) reports the associated normalized mean fluorescence values.

**Figure 2:**
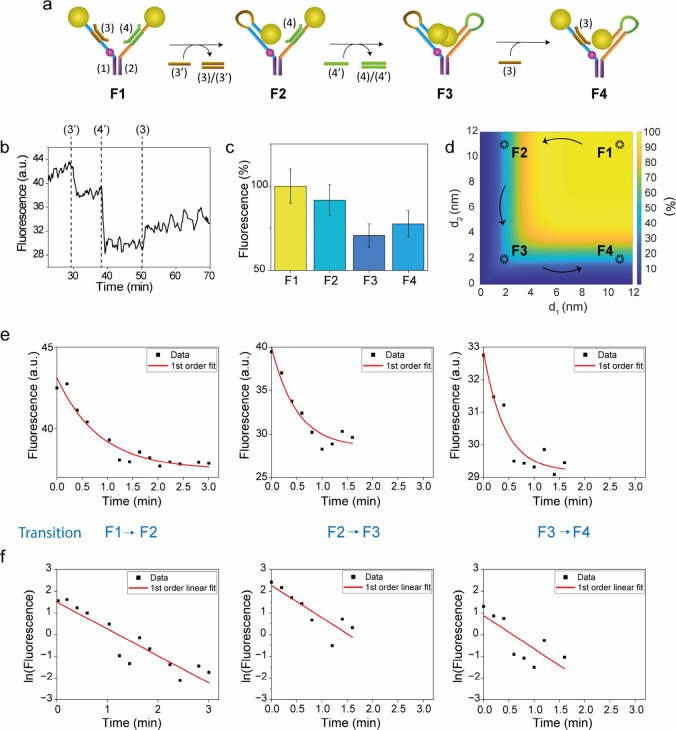
(a) Schematic representation of the transitions across DNA fork states F1, F2, F3, and F4. (b) Fluorescence emission levels monitored at λ = 565 nm associated with the fork reconfigured across states F1 → F2 → F3 → F4 by the sequential addition of strands (3′), (4′), and (3). (c) Bar plot representing normalized mean fluorescence values associated with each state. (d) Bidimensional map of Cy3 theoretical fluorescence quantum yield as a function of its distance from each AuNP d_1_ or d_2_.. (e) Saturation decay-fit system transitions, using the first-order reaction rate law. (f) Single-log plots of the experimental data shown in (e) and their linear fitting (red line) using the integrated first-order reaction rate law (see SI for additional details and fitting parameters).

Fluorescence emission levels are progressively lowered across states F1 → F2 → F3, pointing at the occurrence of increasingly relevant quenching phenomena as the Cy3-AuNP separating distance becomes shorter. State F4 was expected to be perfectly specular to F2, but its associated quenching effect is weaker, likely ascribable to an intrinsic lack of symmetry related to the positioning of Cy3 with respect to the two AuNPs. Since transitions from the completely opened state F1 to partially closed states F2 and F4, and completely closed geometry F3 result in discrete emission levels, each state-associated fluorescence could be described by a set of coordinates derived by the two Cy3-AuNP separation lengths. In Figure 2(d), a bidimensional map of Cy3 theoretical quantum yield (QY) is reported, where separating distances are represented by the vertical and horizontal axes, and increasing QY levels are represented by a dark blue to yellow color-coded surface. Values associated with each system state are highlighted by dashed circles. It is worth mentioning that Cy3 showed no photobleaching during the experiment. To further strengthen this point, a control experiment was conducted to demonstrate no Cy3 photobleaching during the experimental time window. Results are shown in Figure S2, demonstrating fluorophore stability.

To better understand the behavior of the system, kinetic analysis was performed using a standard model. Figure 2(e) reports fluorescence as a function of time for the three individual transitions F1 → F2, F2 → F3, and F3 → F4. Saturation decay trends were interpreted as disappearance of the starting state (e.g. F1 in the F1 → F2 transition), similarly to the consumption of a reactant in a chemical reaction. Experimental points were fitted using both first and second reaction order laws. For details regarding the rate law equations and the resulting *R* ^2^ values, see SI and Table S2. Noteworthy, kinetic data related to the fork system transitions were well interpreted as first-order kinetics. In Figure 2(f), linearized versions of the data shown in Figure 2(e) are represented in semi-log plots. Linear trends were fitted, compatible with first-order kinetic processes such as molecular reconfigurations, further supporting the previous interpretation. Kinetic constants and half-life values are collectively reported in Table 1.

**Table 1:** Reaction Rate Coefficient Values (*k*) and Half-Life Values (*t*_1/2_) of the Fork and Nano-bolas State Transitions

		*k* (min^–1^)		*t*_1/2_ (min)	
Fork transition	Nano-bolas transition	Fork	Bolas	Fork	Bolas
F1→F2	B1→B2	1.2 ± 0.2	0.057 ± 0.003	0.6 ± 0.1	12.2 ± 0.6
F2→F3	B2→B3	1.5 ± 0.3	0.033 ± 0.002	0.5 ± 0.1	21 ± 1
F3→F4	B3→B4	1.5 ± 0.5	0.030 ± 0.002	0.6 ± 0.2	23 ± 1
	B4→B1	-	0.025 ± 0.001	-	27.7 ± 0.9

In the second nanomachine, i.e. “nano-bolas”, a double thiol-modified single-stranded DNA (5), was functionalized with 15 nm AuNPs at the 5′- and 3′-ends, Figure 3(a). Again, this was obtained via thiol-gold chemistry, followed by the isolation of the gold-DNA hybrid product via gel electrophoresis. From the band densities and the disappearance of the reference AuNP band, a ca. 90% yield of AuNP functionalization was estimated. This scaffold was used as a molecular rail for guiding the shift of the shorter Cy3-modified ssDNA (6), acting as a cursor for the modulation of the distance between the fluorophore and the two AuNPs. Figure 3(b) shows the components of the self-assembled system and the final structure comprising double AuNP-functionalized strand (5), Cy3-modified strand (6), and blocking strands (7) and (8). The cursor was programmed to migrate across the scaffold as a response to chemical stimuli, i.e. strand-displacement events between strands (7) and (8) and their respective fully complementary strands (7′) and (8′), to determine the four states B1, B2, B3, and B4. Thanks to sequence complementarity with domain a of the rail, cursor (6) is permanently connected to the AuNP-DNA (5) assembly. All DNA sequences used for nano-bolas are reported in Table S3.

**Figure 3:**
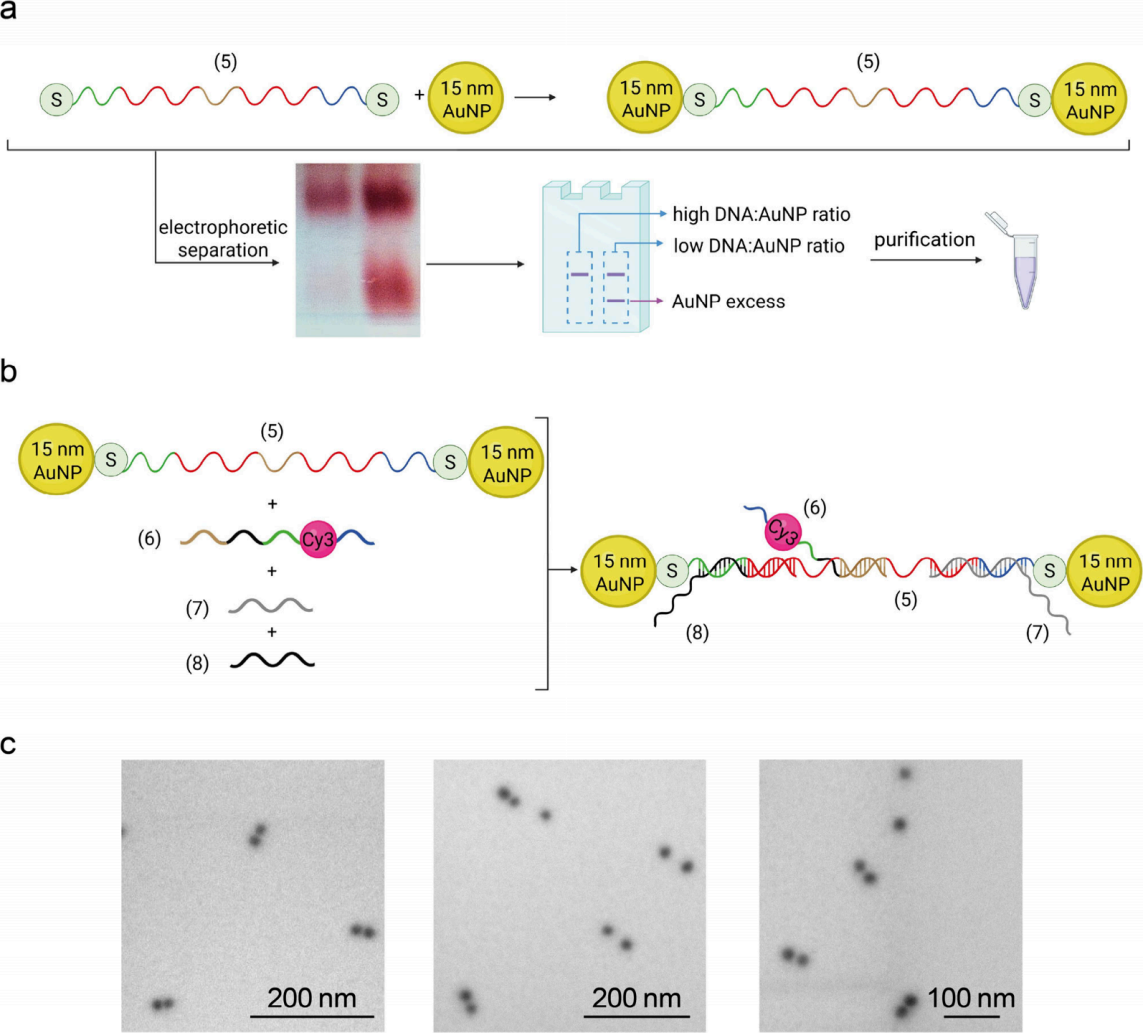
DNA nano-bolas fabrication scheme. (a) Assembly of single DNA-functionalized 15 nm AuNPs and representative agarose gel electrophoretic separation, displaying samples at different thiol-DNA/AuNP molar ratios. One-strand-functionalized particles are collected in a dialysis tube, after band excision from the agarose gel, and cleaned in TBE buffer. (b) Sequence-specific assembly scheme of nano-bolas DNA scaffold; strands (5) and (6) partially interact via complementary domains to form duplex structure (5)/(6), while the remaining ssDNA portions are stabilized by rigidifying strands (7) and (8). (c) Representative STEM micrographs of the synthesized nanoassemblies in state B1.

In Figure 3(c), similarly to the previous nanomachine, STEM morphological characterization of the nanomachine showed pairs of electrondense spherical objects, again pointing at the presence of the DNA scaffold bridging couples of AuNPs.

To trigger the reconfiguration mechanism, a small excess of the appropriate strand was added. A schematic representation of the system evolution across states B1, B2, B3, and B4 is reported in Figure 4(a) (for details of the reconfiguration operations, including volumes and concentrations of all mixtures, see SI). In state B1, domain a, in the middle of the rail, is occupied by the cursor by formation of a partial duplex, while additional domains b and c form two more duplex regions with Cy3-carrying strand (6). In this state, the two AuNPs are positioned at the shortest distance to the fluorophore (ca. 2 nm) and the system is in the closed state. When ssDNA (7) is added to the solution, it behaves as a blocking strand and occupies domain b, positioned between the cursor and the tethered AuNP, by formation of a more stable duplex with strand (5). The system is now in state B2, with strand (6) linked to strand (5) by duplexes on domain a and b and Cy3-AuNP distances d_1_, d_2_ equal to ca. 2 and 20 nm, respectively. To transition to the fully open state B3, ssDNA (8) is added to the solution, releasing (6) from the scaffold domain b. Both Cy3-AuNP distances are now similar and calculated to be ca. 10.5 nm. The last state, B4, is realized by adding (7′), which releases strand (7) forming duplex (7)/(7′) and regenerates the link to domain c. In this state, Cy3-AuNP distances d_1_, d_2_ are symmetric to state B2 (i.e. ca. 20 and 2 nm). Finally, initial state B1 is recovered by adding (8′), which removes (8) by formation of duplex (8)/(8′), realizing the configuration where (6) is linked to (5) by all three domains a, b, and c. A representative two-cycle fluorescence characterization experiment is reported in Figure 4(b), along with state-associated average emission levels in Figure 4(c), and the color-coded 2D QY map relating AuNP-Cy3 calculated distances d_1_, d_2_ with Cy3 theoretical emission in Figure 4(d). It is worth mentioning that fluorescence intensities associated with the second cycle of reconfigurations were almost identical to the first cycle, highlighting the full reversibility of the system and the absence of Cy3 photobleaching during the experimental time interval. This absence of photobleaching is consistent with experimental results of the fork nanomachine (*vide supra*).

**Figure 4:**
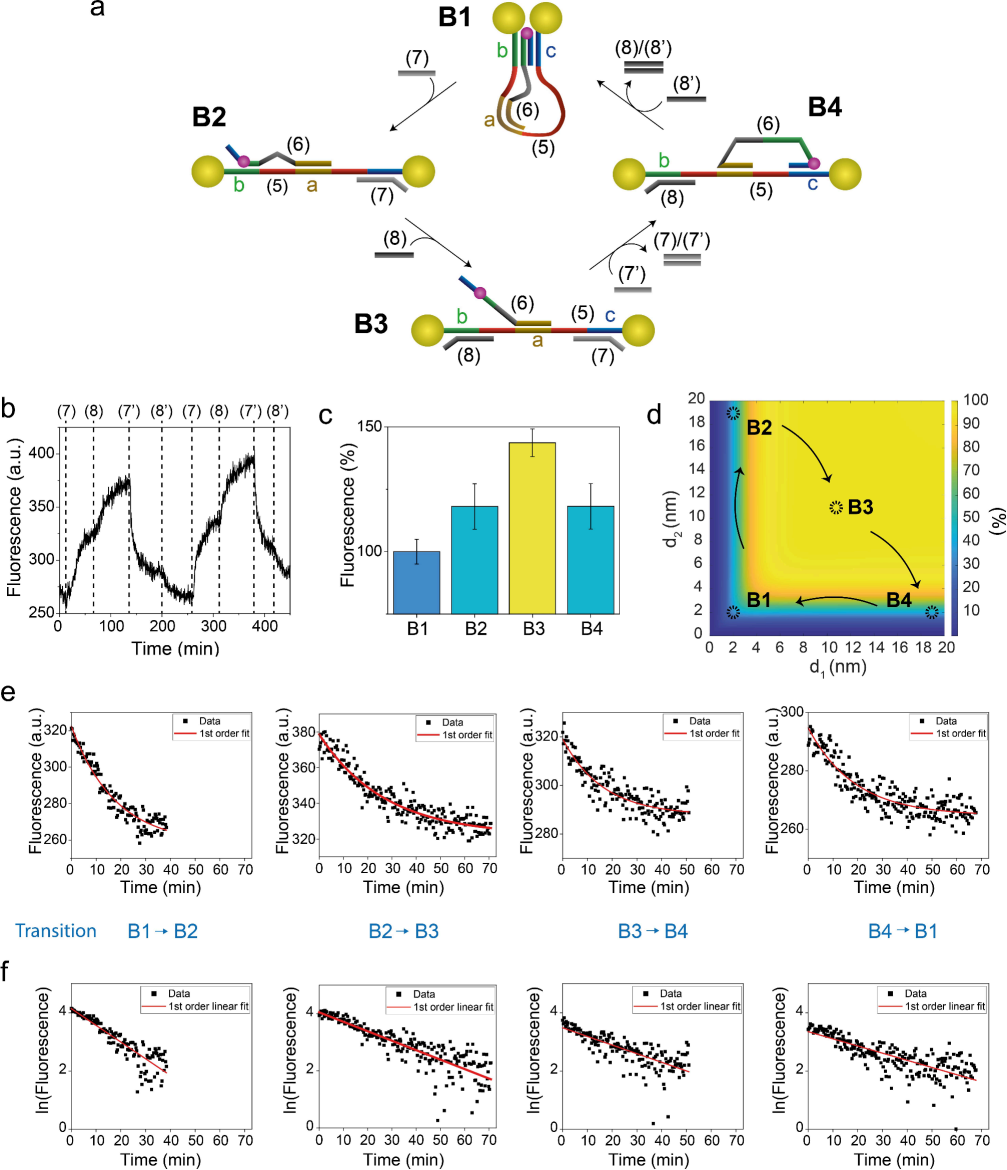
Nano-mechanical control of the fluorescence properties of nano-bolas through strand-displacement reactions. (a) Schematic representation of the transitions across configurations B1 → B2 → B3 → B4. (b) Fluorescence emission levels monitored at λ = 565 nm associated with the system reconfigured across states B1 → B2 → B3 → B4 → B1 → B2 → B3 → B4 → B1. (c) Bar plot representing mean fluorescence values, with the relative standard deviation, for each system state. (d) Map of the theoretical fluorescence quantum yield of Cy3 as a function of its distance from each AuNP. The QY is color coded from dark blue (low QY) to yellow (high QY), and distances are marked in the horizontal and vertical axes. (e) Fittings of the bolas system first cycle transitions across states B1 → B2 → B3 → B4 → B1, using the rate law of a first-order reaction (exponential decay) and (f) using the integrated form in a single-log plot (see also SI for the rate equations, *R* ^2^ parameters, and additional details).

Similarly to the previous system, fluorescence kinetics were fitted using first- and second-order reaction rate laws, with the first-order law showing a better approximation, Figure 4(e). See also the SI for second-order kinetics fittings, *R* ^2^ values, and slope and half-life values of second cycle bolas state transitions (Table S4and S5). Hence, bolas system transitions were assumed to behave as a first-order reaction. Reconfiguration half-life values, expressed in minutes, were calculated and are collectively reported in Table 1. It appears immediately evident that transitions B1 → B2 and B4 → B1 are characterized by opposite behaviors: While the first is very short, the second transition takes a rather longer time. Intuitively, this can be explained by the electrostatic repulsion and steric hindrance of the two AuNPs. While the first transition (opening of the system) takes advantage of the electrostatic repulsion, the B4 → B1 transition must overcome the repulsion, moving the particles from the open to the close configuration. The intermediate transitions, on the other hand, show similar rates, since the interparticle distance does not change during B2 → B3 and B3 → B4 transitions.

In the two dynamic AuNP-DNA hybrid scaffolds, strand-displacement-operated reconfigurations produced discrete Cy3-AuNP separating distances in the 2–20 nm interval that modulated fluorescence emission as reported in Figures 2 and 4. These experimental results are in line with previously reported systems and were further supported by a geometry-based theoretical study, where individual distances between particles and fluorophore were calculated as pairs associated with each state.^46,47^ Distance pairs were calculated by assuming an associated DNA duplex rigid geometry and a base-pair length of 0.34 nm. For simplicity, single-strand portions were assumed to contribute with a similar length. For states F1, F2, F3, and F4 calculated separating distance pairs are 11–11 nm, 11–2 nm, 2–11 nm, and 2–2 nm, respectively, while states B1, B2, B3, and B4 associated distances were calculated to be 2–2 nm, 2–19 nm, 10.5–10.5 nm, and 19–2 nm, respectively. These values were used to theoretically estimate the distance-dependent effect of the plasmonic quencher using an established model.^48^ Results of this analysis are reported in QY color maps of Figures 2(d) and 4(d), showing good agreement with the experimental results. Furthermore, Table 1 collectively reports the kinetic constants and half-time values of the fork and the nano-bolas systems which were obtained from fluorescence kinetics fittings.

To conclude, two DNA-scaffolded dynamic AuNP fluorescent reporters were presented. The general principle behind the design of such responsive systems was the confinement of a fluorophore within a short distance from one or two AuNPs. Both mechanisms successfully operated fine-tuning of the distance separating two 15 nm AuNPs and Cy3, with a quenching effect on Cy3 fluorescence that generated discrete signal outputs. Furthermore, kinetic modeling, which is a relevant aspect for sensing/reporting platforms,^49^ showed good agreement with first-order kinetics, indicating that they share a desired milieu responsiveness. A substantial difference between the emission signals and the reconfiguration kinetics of the two systems was observed. In particular, the nano-bolas showed a higher signal-to-noise ratio and higher quality saturation kinetics. These features were attributed to the different strategies used in the scaffold design. While the first system (bimolecular) comprises a DNA duplex region that joins two ssDNA oligonucleotides, each carrying a single AuNP, the second system (unimolecular) bridges two AuNPs by an individual ssDNA. These design differences have the following consequences: (i) the bimolecular scaffold, i.e. the nano-fork, is less expensive and the assembly procedure is simpler, requiring established protocols for AuNP-thiolated DNA modifications and electrophoretic separation; (ii) the unimolecular scaffold is more expensive and more complex to assemble, due to DNA double-thiol modifications and assembly optimization; in addition, a lower AuNP functionalization yield was estimated, likely due to the double-thiol modification of the scaffold; (iii) the second scaffold is more stable, since the AuNPs are linked by covalent bonds resulting in higher quality reconfigurations; and (iv) the nano-bolas shows an overall kinetics one order of magnitude slower with respect to the fork. This may be attributed to the fact that transitions in the bolas system require a change of conformation involving a larger number of atoms with respect to the fork system (see Figure 5 for a schematic representation comparing the two systems). If a higher number of internal coordinates is involved (within conformational changes of the same type), then the process is likely to be less kinetically favored. However, one order of magnitude difference in the response kinetics can be regarded of secondary importance when considering that the bolas system displays a better signal-to-noise ratio and a noteworthy reversible fluorescence emission, along with high-quality saturation kinetics. Overall, this makes the nano-bolas system an elective milieu-sensitive nanodevice for small-volume and low-concentration (1 nM AuNPs) detection of single-strand nucleic acid targets, such as viral genetic material or miRNA, potentially applicable as a multiresponsive fluorescent reporter for *in vitro* tests. In addition, fine-tuning of such DNA-based nanoconfinement might lead to a plasmonic hotspot (see an example in Figure S3 and accompanying Table S6), where the correct placement of two AuNPs with respect to the fluorophore can turn the system from plasmonic-quenching to plasmonic-enhancing. This would further improve the reporter mechanism, giving a signal-ON readout for the sensing event.

**Figure 5:**
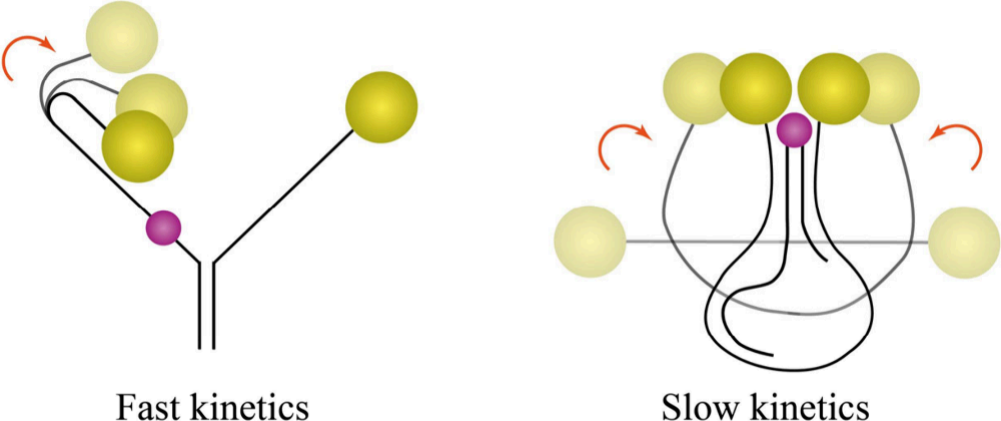
Schematic representations of the nanofork (on the left) and nano-bolas (on the right) transitions across configurations F1 → F2 and B4 → B1, respectively, that emphasize the difference in portions of the structures involved.

## Supplementary Material



## Notes

Table S1. Sequences of the core structure oligonucleotides and of the operating strand for the nanofork system; Table S2. *R* ^2^ values of fluorescence signal fits for the nanofork system; Table S3. Sequences of the core structure oligonucleotide and of the operating strands for the nano-bolas system; Table S4. *R* ^2^ values of fluorescence signal fits for the nano-bolas system; materials and methods for the single and double AuNP-modified oligonucleotides and their stabilization; methods for the fluorescence signals fit with first- and second-order reaction rate laws; methods for the theoretical calculations correlating the distance-dependent fluorescence quantum yields of the Cy3 fluorophore in the here presented milieu-sensitive fluorescent reporters; Figure S1. Fluorescence transitions associated with the second cycle of the nano-bolas system and first-order reaction rate law fits; Figure S2. Cy3 photobleaching control experiment; Table S5. Slope and half-life values of bolas state transitions (second cycle). Table S6. Sequences of the core structure oligonucleotide and of the operating strands for the fluorescence-enhancement plasmonic scaffold; Figure S3. Fluorescence-enhancement DNA-scaffolded plasmonic nanostructure (PDF)

## Abbreviations

AuNP: gold nanoparticle
Cy3: sulfo-cyanine 3
TEM: transmission electron microscopy
STEM: scanning-transmission electron microscopy
QY: quantum yield
ssDNA: single-stranded DNA
