# Supplementary material for: Plasmonic Nano-bolas Hunt DNA Targets
Source: ACS Nanosci Au. 2025 Nov 14;6(1):35–43. doi: 10.1021/acsnanoscienceau.5c00131 (PMC12921593; doi:10.1021/acsnanoscienceau.5c00131)
Supplement: Supplementary file 1 [file ng5c00131_si_001.pdf]

Supplementary Information

Plasmonic Nano-bolas Hunt DNA targets

Aura Cencini[1,†], Graziano Rilievo[1,†], Mirco Zerbetto[2], Mary Bortoluzzi[1], Federica Tonolo[1], Fabio Vianello[1], Alessandro Cecconello[1,3,], and Massimiliano Magro[1]*

1. Department of Comparative Biomedicine and Food Science, University of Padova, Viale dell'Università 16, 35020, Legnaro (PD), Italy

2. Department of Chemical Science, University of Padova, via Marzolo 1, 35131, Padova (PD), Italy

3. Department of Molecular and Translational Medicine, University of Brescia, Viale Europa 11, 25123, Brescia (BS), Italy

† These authors contributed equally to this work

* Correspondence: alessandro.cecconello@unibs.it

1. Materials

Glycerol, Sodium chloride, bis(p-sulfonatophenyl)phenylphosphine dihydrate dipotassium salt (BSPP), and tris(2-carboxyethyl)phosphine hydrochloride (TCEP) were purchased from Sigma–Aldrich (USA). 15 nm gold colloid was purchased from BBI Solutions. “Metaphor” agarose was purchased from Lonza (Switzerland). 10 kDa MWCO “Snakeskin” dialysis tubings were purchased from Thermo Scientific (USA). Amicon ultra 100 kDa MWCO were purchased from Millipore (Germany). 0.22 μm disposable filter units were purchased from Whatman GmbH. Amersham G-25 microspin columns were purchased from GE Healthcare (USA). All DNA oligonucleotides were purchased from Integrated DNA Technologies, IDT (USA). Ultrapure water from a NANOpure Diamond (Thermo scientific, USA) source was used in all experiments. 10X Tris-Borate-EDTA (TBE) buffer was purchased from Bio-Lab ltd (Israel). Amersham microspin G-25 columns were purchased from Cytiva.

2. Pre-functionalization of commercial AuNPs and preparation of dithiol DNA stock solution

15 nm AuNPs, as provided by the manufacturer, were mixed with an excess of Bis(p-sulfonatophenyl)phenylphosphine dihydrate dipotassium salt (BSPP) and stirred for 8 hours. The resulting AuNPs were, then, concentrated using Amicon filtering tubes 100 kDa MWCO, for 5 min under 3000g to a final concentration of 1-10 μM (if necessary, a second concentration step was performed using Amicon filtering tubes). AuNP concentration was estimated by diluting the resulting solution (1:1000), recording the absorption spectrum, and using AuNP extinction coefficient at 520 nm. The resulting AuNP solution was stored at +4°C and used for several days.

Dithiol-functionalized DNA strands were diluted to a final concentration equal to 15 μM with 15 mM Tris(2-carboxyethyl)phosphine hydrochloride (TCEP). DNA solutions were stored at -20°C and used for several weeks. Prior to each use, AuNPs were subjected to desalting to remove excess TCEP using Amersham microspin G-25 columns, following manufacturer instructions.

3. Conjugation of AuNPs and thiolated nucleic acid strands

DNA-functionalized AuNP were prepared as follows: Thiolated strands as prepared in point 2, were mixed in a solution that included 0.5X TBE, 0.6 mg/mL BSPP, and 100 mM NaCl. The resulting mixture was incubated with 15 nm AuNPs, as prepared in point 2. The mixture was, then, incubated for 2 hours at room temperature lo allow conjugation of the thiol to the gold surface and then separated by gel electrophoresis (see next paragraph, point 4).

4. Purification of the DNA-functionalized AuNPs

The procedure for obtaining one- or two-oligonucleotide functionalized AuNP was already described in detail elsewhere[1–3]. Here, a short version of the protocol is reported. Single DNA-functionalized AuNPs were separated from the polyconjugated DNA-AuNP mixture, and the unreacted AuNPs, on a 3% w/v agarose gel (MetaPhor). Samples were mixed with 12 % v/v glycerol (final concentration) and loaded into the gel wells. Gels were run at a constant voltage of 70 V in an ice bath (ca. 10°C). When satisfactory separation was observed, bands of interest were excised and subjected to a voltage of 100 V inside a closed Snakeskin dialysis membrane (10kDa MWCO) filled with 0.5X TBE buffer (to release the desired modified NPs from the agarose). Then, the solution trapped in the dialysis membrane was collected, filtered with a 0.22 µm syringe filter (Whatman), and concentrated with a 100 kDa MWCO Amicon filtering tube.

5. Stabilization of the single DNA-functionalized AuNPs

The separated DNA-functionalized AuNPs displayed limited stability in water and precipitated with time. To eliminate this difficulty, the hybrids were stabilized with an excess of a short thiolated DNA oligo (HS-TTTTT, Integrated DNA Technologies) in a solution that included 100 mM NaCl, 0.5X TBE and 0.6 mg/mL BSPP. The resulting mixture was incubated at 25°C for 48 h and then washed four times using 100 kDa MWCO Amicon filtering tubes 5 min × 3000g, while diluting the samples to 15 mL in water each time, to remove stabilizer excess. At the end, the stabilized DNA-functionalized AuNPs were diluted, and their concentration was determined by recording the absorption spectrum.

6. Assembly and operation of the nano-devices.

Stabilized DNA-conjugated AuNPs were diluted to a final concentration of 1 nM in 500 mM NaCl and TBE 0.5X. Reconfigurations were operated directly in a quartz cuvette (3 mm light path) at a nano-device concentration of 0.5 nM, while recording fluorescence emission at 565 nm. Stepwise additions of the reconfiguration strands were carried out with volumes equal to 0.5 μL and concentrations were adjusted to obtain a final dilution equal to 100 nM.

7. STEM characterization

The two nanostructures were prepared for STEM analyses in a solution containing 0.4 mg/mL BSPP, 0.1X TBE, 700 mM NaCl. The resulting nanostructures were diluted to a final concentration of 1 nM in a 200 mM NaCl solution and deposited on copper grids. The drop was dried in air at room temperature for two hours. Images were recorded with an Extra High-Resolution Scanning-Transmission Electron Microscope (XHR-STEM) Magellan 400L (FEI – Thermo Fisher Scientific) with the scanning-transmission electron microscopy detector. Settings: 25-27 kV, 0.1-0.2 nA.

8. Theoretical calculations correlating distance-dependent Cy3 fluorescence quantum yields in the AuNP-functionalized structures.

Theoretical calculation correlating the distance-dependent fluorescence emission yields of the Cy3 fluorophore in the AuNP-DNA hybrids were simulated theoretically by using the classical model proposed by Gersten and Nitzan, adapted for emitters at a given distance from the surface of a spherical metal nanoparticle.[4] The calculation of the radiative and non-radiative rates for the emitter-nanoparticle system has been carried out with an in-house written Python script (Python implementation is available for download at https://github.com/mircozerbetto-unipd/Gersten-Nitzan_QY/tree/main). Parameters of the model are: Nanoparticle diameter 15 nm, wavelength 650 nm, medium refractive index 1.33, quantum efficiency of the emitter 0.01, cutoff for the multipole expansion: 60. The dielectric function of the Au nanoparticle (ϵ_{Au}) as function of the frequency (ω) and the nanoparticle radius (R) has been calculated as[5]:

$$\epsilon_{\mathrm{Au}}(\omega, R) = \epsilon_{\mathrm{Au},\infty} - \frac{\omega_{\mathrm{p}}}{\omega^2 + i\left(\gamma_{\mathrm{bulk}} + C\frac{v_{\mathrm{F}}}{R}\right)\omega} + \frac{A}{1 + \exp(-(\omega - \omega_{\mathrm{c}})/\Delta)} \tag{1}$$

with $\epsilon_{\mathrm{Au},\infty} = 9.84$, $\omega_{\mathrm{p}} = 9.01$ eV, $\gamma_{\mathrm{bulk}} = 0.072$ eV, $C = 0.33$, $v_{\mathrm{F}} = 1.4 \cdot 10^6$ m/s, $A = 5.6$, $\omega_{\mathrm{c}} = 2.4$ eV, and $\Delta = 0.17$ eV.[46]

The quenching effect on Cy3 due to the interaction with two nanoparticles has been estimated by considering the single-nanoparticle radiative and non-radiative rates as additive, which is a good approximation for interparticle distances larger than 5 nm.[6]

9. 1st or 2nd order reaction kinetics fitting of the fluorescence data.

Cy3 fluorescence signal, for both fork and nano-bolas systems, was considered in the time intervals associated with individual transitions. Each time interval was shifted so that the initial time was set at 0 minutes, and all signals were converted to decreasing curves, to use first or second order reaction equations considering the starting state of the transition the "reactant" consumed during the reaction. To verify if fluorescence kinetics, for each state transition, were following a first or second order reaction rate law, a fitting was performed for each order. As first order reaction rate law, the following equation (2) was used:

$$f(t) = (f_1 - f_0)e^{-kt} + f_0 \tag{2}$$

Where t is time (in minutes), $f(t)$ is Cy3 fluorescence in time, f_1 represents the initial fluorescence value, f_0 represents the final fluorescence value (or minimum fluorescence), and k is the reaction rate constant (in min^{-1}).

The second order reaction rate law was set as follows:

$$f(t) = \frac{1}{\frac{1}{f_1 - f_0} + kt} + f_0 \tag{3}$$

Where t is time (in minutes), $f(t)$ is Cy3 fluorescence in time, f_1 represents the initial fluorescence value, f_0 represents the final fluorescence value (or minimum fluorescence), and k is the reaction rate constant (in min^{-1}).

Since all fluorescence curves were better approximated by a first order rate law, i.e. the R^2 value resulted to be higher, the natural logarithm of fluorescence signals, subtracted of their minimum

value, was computed for each transition and a linear fit was performed. Linear curves were computed according to the equation of a first order reaction, written in its integrated form:

$$y = -k \cdot t + \ln(y_0) \qquad (4)$$

Where y is the natural logarithm of the fluorescence signal, t is time (in minutes), and y_0 is the fluorescence value for t = 0 min. All data analyses and fittings were performed with OriginLab software.

10. Fabrication and operation of intermolecular plasmonic scaffold control

Cy3-modified single strand DNA (10) in the absence of blocking strands (11) and (12) was subjected to individual 15 nm gold nanoparticles functionalized with thiolated single-stranded DNA (13) and (14), producing self-assembled structure shown in Figure S3a. Nanoparticle functionalization and purification was carried out according to the procedure already described in previous paragraphs. The control structure was disassembled using strands (11') and (12'), resulting in fluorescence emission levels reported in Figure S3b.

Table S1. Sequences of fork core structure oligonucleotides and operating strands (5' -> 3')

(1)	GTT GTG TCC TAT TAT GG - Cy3 – T CGG CTC CTA AAT CTG AAC TGT ATG CGT AAA GGA GC-$(SH)_2$
(2)	$(SH)_2$- CGC AGT CTT GGT CTC GAA ATC TTG GTA ACT GCG CTG GAG TAA TAG GAC ACA AC
(3)	ATA CCT GCC TTT ACG CAT ACA GTT CAG ATT TAG GAT ATA GCG
(3')	CGC TAT ATC CTA AAT CTG AAC TGT ATG CGT AAA GGC AGG TAT
(4)	TCT CTT TCA GTT ACC AAG ATT TCG AGA CCA AGA CTG AAC ATG
(4')	CAT GTT CAG TCT TGG TCT CGA AAT CTT GGT AAC TGA AAG AGA

Table S2. R^2 values resulting from fittings of fluorescence saturation kinetics of the nano-fork system with a first or second order rate law (eq. 2 and 3, respectively)

Transition	R^2 first order rate law fit	R^2 second order rate law fit
F1→F2	0.95	0.29
F2→F3	0.94	0.49

F3→F4	0.91	0.90

Table S3. Sequences of bolas core structure oligonucleotides and operating strands (5' -> 3')

(5)	$(SH)_2$ -TTT GCA TCT AGT GTG AAGTAT GAT CCT GTG CTC GTC TGA CGC ATA CGA AGT TCT CAT TTT-$(SH)_2$
(6)	GAC GAG CAC AGG ACT GTG ACT AGA TGC - Cy3 -TTT ATG AGA ACT AGA CTG
(7)	CGT TCG CAG AAT TGG G AAT GAG AAC TTC GT ATG CGT
(7')	ACG CAT ACG AAG TTC TCA TTC CCA ATT CTG CGA ACG
(8)	CTT CAC ACT AGA TGC AAC GTA TAA GCT CTG
(8')	CAG AGC TTA TAC GTT GCA TCT AGT GTG AAG

Table S4. R^2 values resulting from fittings of fluorescence saturation kinetics of the nano-bolas system with a first or second order rate law (eq. (2) and (3), respectively)

Transition	R^2 first order rate law fit	R^2 second order rate law fit
B1→B2 (1st cycle)	0.93	0.04
B2→B3 (1st cycle)	0.90	0.01
B3→B4 (1st cycle)	0.79	0.04
B4→B1 (1st cycle)	0.83	0.02
B1→B2 (2nd cycle)	0.89	0.04
B2→B3 (2nd cycle)	0.81	0.02
B3→B4 (2nd cycle)	0.91	0.06
B4→B1 (2nd cycle)	0.82	0.03

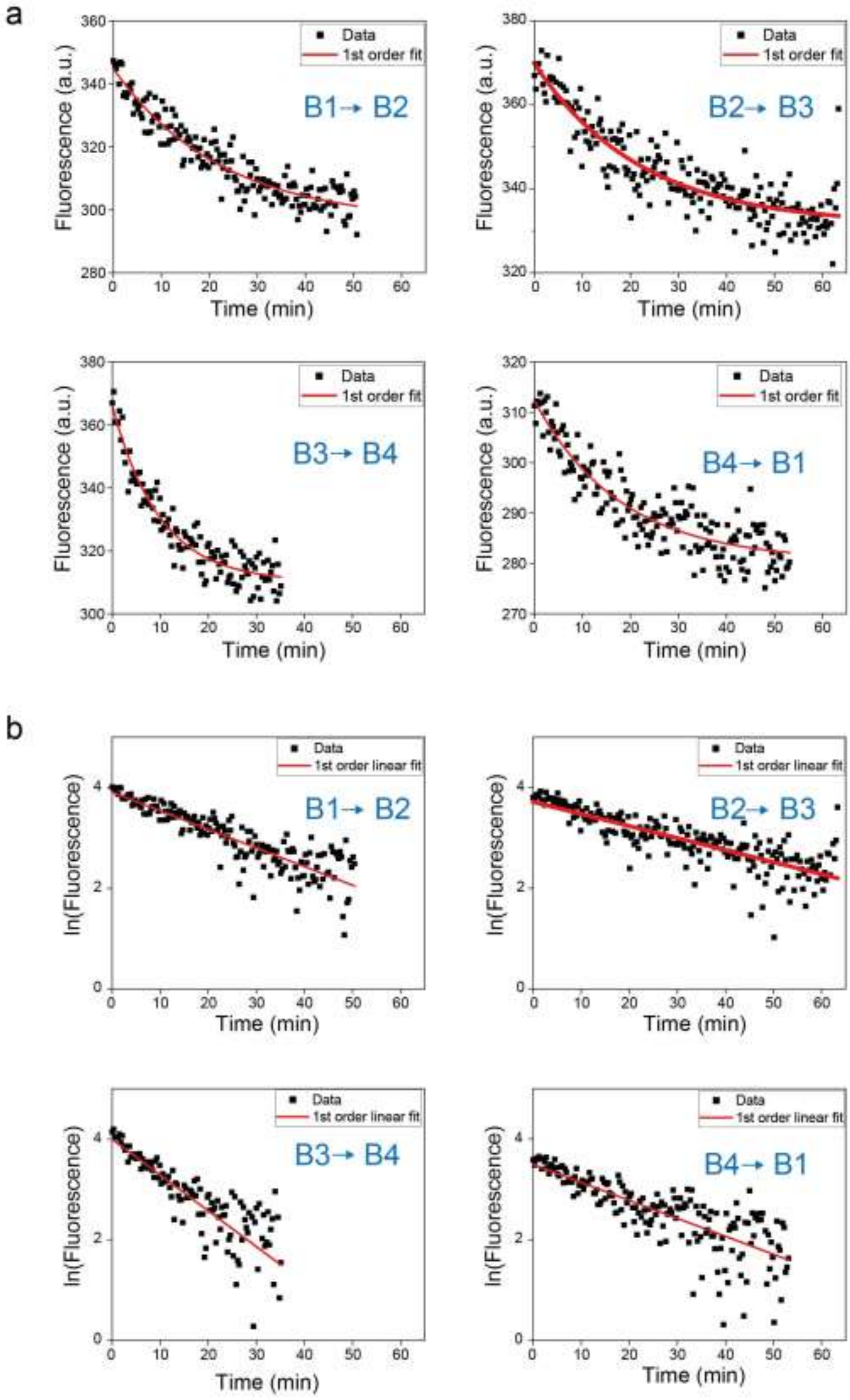

Figure S1. Fluorescence characterization of the nano-bolas system second cycle transitions, across reconfiguration states, along with the rate law fit of a first-order reaction in its exponential (a) and in its linear form (b).

Table S5. Slope values (k) and half-life values ($t_{1/2}$) of nano-bolas state transitions (second cycle).

Transition	k (min^{-1})	$t_{1/2}$ (min)
1	0.037	18.73
2	0.024	28.88
3	0.071	9.76
4	0.036	19.25

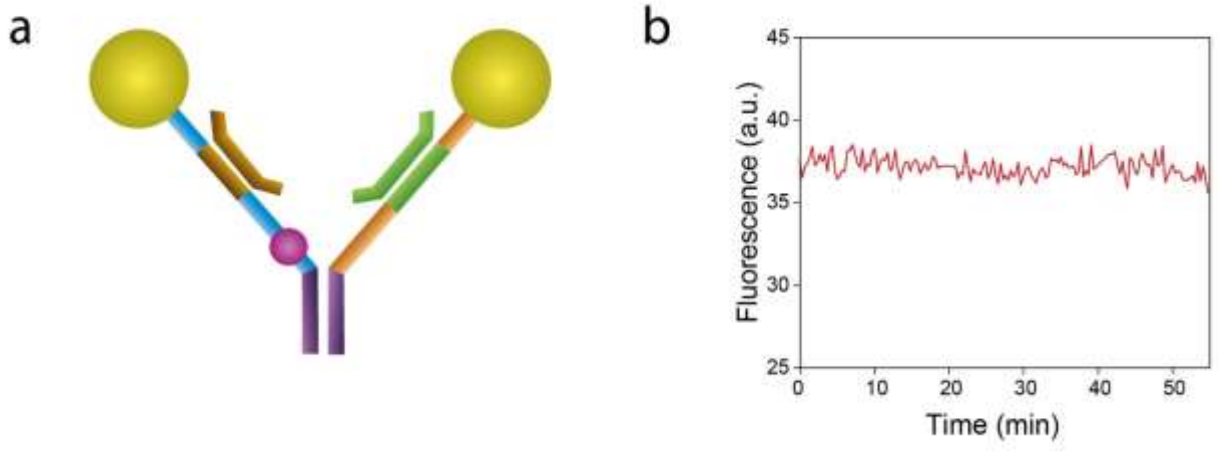

Figure S2. Control experiment of the fork in the F1 configuration (a) without reconfigurations, showing a fluorescence signal where no appreciable photobleaching is detected (b).

Table S6. Sequences of control core structure oligonucleotides and operating strands (5' -> 3')

(10)	GATCACGT TGA GTC TTG TGT A GA TTG TCG C –Cy3- CA GTGCTT A GG ATGCTT CTC GTG TGTCCAA
(11)	CTACACAAGACTCAACGTGATCTTAGTTAGTTAGTTAG
(11')	CTAACTAACTAACTAAGATCACGTTGAGTC
(12)	ACTACATACATACATACTTGGACACACGAGAAGCATCC
(12')	CTC GTG TGT CCA AGT ATG TAT GTA TGT AGT
(13)	$(SH)_2$- TTTTGCG ACA ATCGCCTGCTAC ACA AGA CTC AAC G
(14)	CAC ACG AGA AGC ATC C CGCTCGTA AGC ACT GTTTT –$(SH)_2$

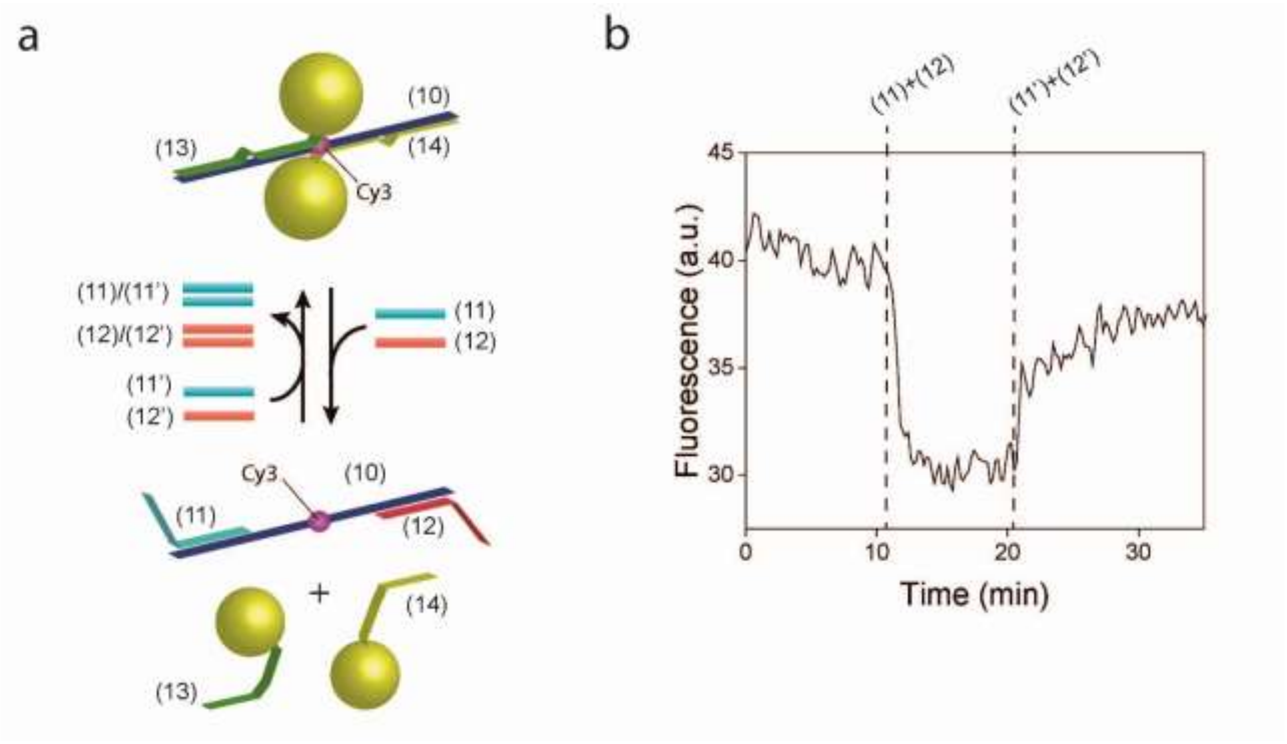

Figure S3. Assembly and disassembly scheme (a) of the control DNA-plasmonic scaffold where fluorophore Cy3 is placed between two 15-nm AuNP or detached from the AuNPs. (b) fluorescence emission recorded for the assembled system, disassembled system upon addition of oligonucleotides (11) and (12), and after recovery of the initial state upon addition of oligonucleotides (11') and (12').

References

1. Elbaz, J.; Cecconello, A.; Fan, Z.; Govorov, A.O.A.O.; Willner, I. Powering the Programmed Nanostructure and Function of Gold Nanoparticles with Catenated DNA Machines. *Nat Commun* **2013**, *4*, 2000.

2. Cecconello, A.; Lu, C.H.C.-H.C.-H.; Elbaz, J.; Willner, I. Au Nanoparticle/DNA Rotaxane Hybrid Nanostructures Exhibiting Switchable Fluorescence Properties. *Nano Lett* **2013**, *13*, 6275–6280.

3. Shimron, S.; Cecconello, A.; Lu, C.H.C.-H.C.-H.; Willner, I. Metal Nanoparticle-Functionalized DNA Tweezers: From Mechanically Programmed Nanostructures to Switchable Fluorescence Properties. *Nano Lett* **2013**, *13*, 3791–3795.

4. Mertens, H.; Koenderink, A.F.; Polman, A. Plasmon-Enhanced Luminescence near Noble-Metal Nanospheres: Comparison of Exact Theory and an Improved Gersten and Nitzan Model. *Phys Rev B Condens Matter Mater Phys* **2007**, *76*.

5. Derkachova, A.; Kolwas, K.; Demchenko, I. Dielectric Function for Gold in Plasmonics Applications: Size Dependence of Plasmon Resonance Frequencies and Damping Rates for Nanospheres. *Plasmonics* **2016**, *11*.

6. Sánchez-González, Á.; Corni, S.; Mennucci, B. Surface-Enhanced Fluorescence within a Metal Nanoparticle Array: The Role of Solvent and Plasmon Couplings. *Journal of Physical Chemistry C* **2011**, *115*.
